# L-Lactic Acid-Based N-Doped Carbon Quantum Dots with Phenylenediamine Isomers as a Nitrogen Source for the Highly Sensitive Detection of Fe^3+^ Ions

**DOI:** 10.3390/ma19122481

**Published:** 2026-06-10

**Authors:** Ruizhe Wang, Xuanxuan Wang, Dongxia Han, Yaling Zhou, Qinwei Gao

**Affiliations:** 1 College of Chemical Engineering, Nanjing Forestry University, Nanjing 210037, China; wrz_njfu@njfu.edu.cn (R.W.); 15098299627@njfu.edu.cn (X.W.); 8240210157@njfu.edu.cn (D.H.); 18609617692@163.com (Y.Z.); 2 Jiangsu Key Laboratory for the Chemistry and Utilization of Agricultural and Forest Biomass, Nanjing Forestry University, Nanjing 210037, China

**Keywords:** lactic acid, carbon quantum dots, fluorescence sensing, phenylenediamine

## Abstract

Three kinds of nitrogen-doped carbon quantum dots (N-CQDs) were successfully fabricated through a one-pot hydrothermal reaction at 180 °C for 12 h. L-lactic acid served as the carbon precursor, while three phenylenediamine isomers (o-phenylenediamine, m-phenylenediamine, p-phenylenediamine) were employed as nitrogen dopants, yielding samples denoted as OPD-LA, MPD-LA, and PPD-LA. All as-prepared N-CQDs presented uniformly dispersed spherical nanostructures, with average particle sizes of 8.2 nm (OPD-LA), 9.3 nm (MPD-LA), and 10.5 nm (PPD-LA). Abundant surface functional groups, including hydroxyl, carboxyl, amino, and amide moieties, endowed these N-CQDs with outstanding water solubility and tailorable fluorescence emission. The maximum emission wavelengths were centered at 550 nm, 505 nm, and 450 nm for OPD-LA, MPD-LA, and PPD-LA, respectively, exhibiting excitation-independent emission positions yet excitation-dependent intensity. MPD-LA delivered the highest fluorescence quantum yield of 9.59%, and the incorporation of lactic acid significantly elevated the quantum yield of all samples. The N-CQDs maintain high fluorescence intensity and favorable stability within the pH range of 4–11, possessing outstanding salt resistance and stable storage performance for six months. Their fluorescence was effectively quenched upon exposure to Fe^3+^, with a linear detection range of 10–100 μM and a low limit of detection (*LOD*) of 1.49 μM. These lactic acid-derived N-CQDs hold great promise as functional fluorescent probes for Fe^3+^ sensing applications.

## 1. Introduction

In the 21st century, nanomaterials have become one of the most dynamic research frontiers in materials science owing to their unique structural features and exceptional performance, driving technological innovations and large-scale applications across energy, catalysis, and biomedicine [1]. As a typical representative of zero-dimensional nanomaterials, carbon quantum dots (CQDs) have rapidly become a research focus in the field of nanomaterials owing to their advantages, including excellent optical emission properties [2], low cytotoxicity [3], superior water solubility, and good biocompatibility [4]. The discovery of CQDs can be traced back to 2004, when Xu et al. [5] accidentally isolated a novel fluorescent nanomaterial, namely CQDs, during the experiment of purifying single-walled carbon nanotubes by arc discharge. Typically composed of carbon, oxygen, and hydrogen, CQDs possess a spherical or near-spherical morphology with an average diameter of approximately 10 nm. Its internal structure is centered on sp^2^-hybridized carbon atoms, while the outer part consists of sp^3^-hybridized carbon atoms [6], and the surface is rich in functional groups such as carboxyl and hydroxyl groups [7,8].

In terms of synthesis and preparation, researchers have developed a variety of carbon sources and synthetic processes suitable for CQDs. Carbon sources include natural or synthetic materials such as graphite [9], citric acid [10], watermelon peel [11], and white ginger juice [12], while synthetic methods include hydrothermal method [13,14], arc discharge method [15], microwave-assisted synthesis [16,17], laser ablation method [18,19], ultrasonic synthesis [20], chemical oxidation method [21] and electrochemical method [22,23,24]. CQDs synthesized via these approaches typically possess surface-active functional moieties including amino, hydroxyl, and carboxyl groups, which are introduced during the preparation process. These functional groups endow CQDs with excellent optical properties, favorable biocompatibility and water solubility, as well as low cytotoxicity. Meanwhile, the overall preparation process is simple and cost-effective. These characteristics promote the expansion of CQDs’ applications in energy storage, drug delivery, biosensing, bioimaging, and other fields.

However, several technical problems remain to be urgently solved in the current preparation processes of CQDs. The core issue lies in the insufficient controllability of the synthesis process, resulting in common defects of low quantum yield and insufficient purity in the prepared products. Meanwhile, the problem of a wide particle size distribution leads to a broad peak feature in the emission spectra of CQDs, which limits their application in high-precision optical devices and biological detection. To address these problems, existing studies mainly optimize the synthesis by strategies such as utilizing new carbon sources, regulating the reaction solvent and temperature, as well as introducing heteroatom doping with nitrogen, sulfur, and other elements.

Lactic acid [25] and its polymer polylactic acid (PLA) [26], as renewable carbon source materials, exhibit remarkable application advantages. Lactic acid can be prepared via the fermentation of natural biomass such as sugar beet roots, leaves, and plant roots, while polylactic acid is synthesized from lactic acid monomers and possesses characteristics including biodegradability, excellent biocompatibility, and low toxicity [27]. From the perspective of molecular structure, both lactic acid and polylactic acid contain two functional groups, namely carboxyl and hydroxyl groups, which provide abundant active sites for the preparation of CQDs, making them promising carbon source materials [28]. Using lactic acid or polylactic acid as carbon sources for the preparation of CQDs not only provides a new regulatory dimension for tuning the optical properties and structural characteristics of CQDs (e.g., regulating CQDs’ performance through the molecular weight of polylactic acid) but also establishes a novel strategy for the high-value utilization of polylactic acid-based materials [29]. Meanwhile, it provides a brand-new research route for enhancing the fluorescence performance of CQDs and tackling the key bottlenecks in current technologies.

L-Lactic acid, a widely used bio-based platform chemical, contains both hydroxyl and carboxyl functional groups. In parallel, phenylenediamine carries abundant amino moieties. These structural features render them ideal precursors for fabricating N-CQDs. In this work, N-CQDs were fabricated through a one-pot hydrothermal method, with L-lactic acid serving as the carbon precursor and three phenylenediamine isomers acting as separate nitrogen dopants. The resultant N-CQDs are rich in surface functional groups and present outstanding water dispersibility and favorable fluorescence characteristics. Their morphological features, surface chemical structures, and optical properties were fully investigated and analyzed. Different from our previous work, where the particle size ranged from 1 to 4 nm, the samples in this study have a particle size of 8–10.5 nm. The steric hindrance of phenylenediamine isomers can regulate the particle size and optical properties. Upon excitation with 365 nm ultraviolet light, the N-CQDs emit yellow, turquoise and blue fluorescence, respectively. Meanwhile, the resultant N-CQDs present a satisfactory detection linear range and sensing sensitivity toward Fe^3+^ ions, which reveals their great application potential as fluorescent sensing platforms for Fe^3+^ detection. Different from our previous research on lactic acid-based carbon quantum dots (CQDs) focusing on basic synthesis and properties, the present work adopts three phenylenediamine isomers as nitrogen sources to fabricate a series of N-CQDs. We systematically compare their optical performances and further explore the potential of these materials for fluorescent detection of Fe^3+^, which broadens the application scope of lactic acid-derived CQDs.

## 2. Materials and Methods

### 2.1. Materials

L-lactic acid was acquired from Musashino Chemical (Yichun, China) Co., Ltd. Poly(L-lactic acid) was synthesized in our own laboratory. Iron(III) chloride hexahydrate (A.R. Nanjing, China), o-phenylenediamine (oPD, A.R. Nanjing, China), m-phenylenediamine (mPD, A.R. Nanjing, China), p-phenylenediamine (pPD, A.R. Nanjing, China), quinine sulfate (A.R. Nanjing, China), sodium hydroxide (A.R. Nanjing, China), hydrochloric acid (A.R. Nanjing, China), and sodium chloride (A.R. Nanjing, China) were all obtained from Nanjing Chemical Reagent Co., Ltd. (Nanjing, China). All reagents were of analytical grade and used directly without further purification.

### 2.2. Characterization Methods

UV-Vis absorption spectra were collected using a UV-1800PC spectrophotometer (MAPADA, Shanghai, China). For UV-Vis and fluorescence tests, N-CQDs were first dispersed in ethanol to form a 0.05 mg/mL aqueous dispersion. Fourier-transform infrared (FTIR) spectra were recorded on an FTIR-650 spectrometer (GangDong, Tianjin, China) over a wavenumber range of 450–4000 cm^−1^ with KBr pellets as the testing medium. X-ray photoelectron spectroscopy (XPS) was carried out on an AXIS UltraDLD system (Shimadzu, Kyoto, Japan) to analyze the elemental composition and chemical valence states. Fluorescence spectra were acquired on a PerkinElmer FL6500 fluorescence spectrometer (PerkinElmer, Shelton, CT, USA) with an excitation slit width of 5 nm, an emission slit width of 5 nm, and a scanning rate of 1200 nm/min. The particle size and dispersion distribution of N-CQDs were determined using a Zetasizer Nano-ZS (Malvern Instruments, Worcestershire, UK). High-resolution transmission electron microscopy (HRTEM) observations were performed on a JEM-2100 microscope (JEOL, Akishima, Japan) to characterize the microstructure and morphology of the as-prepared N-CQDs.

### 2.3. Synthesis of Nitrogen-Doped Carbon Quantum Dots

**Scheme 1:**
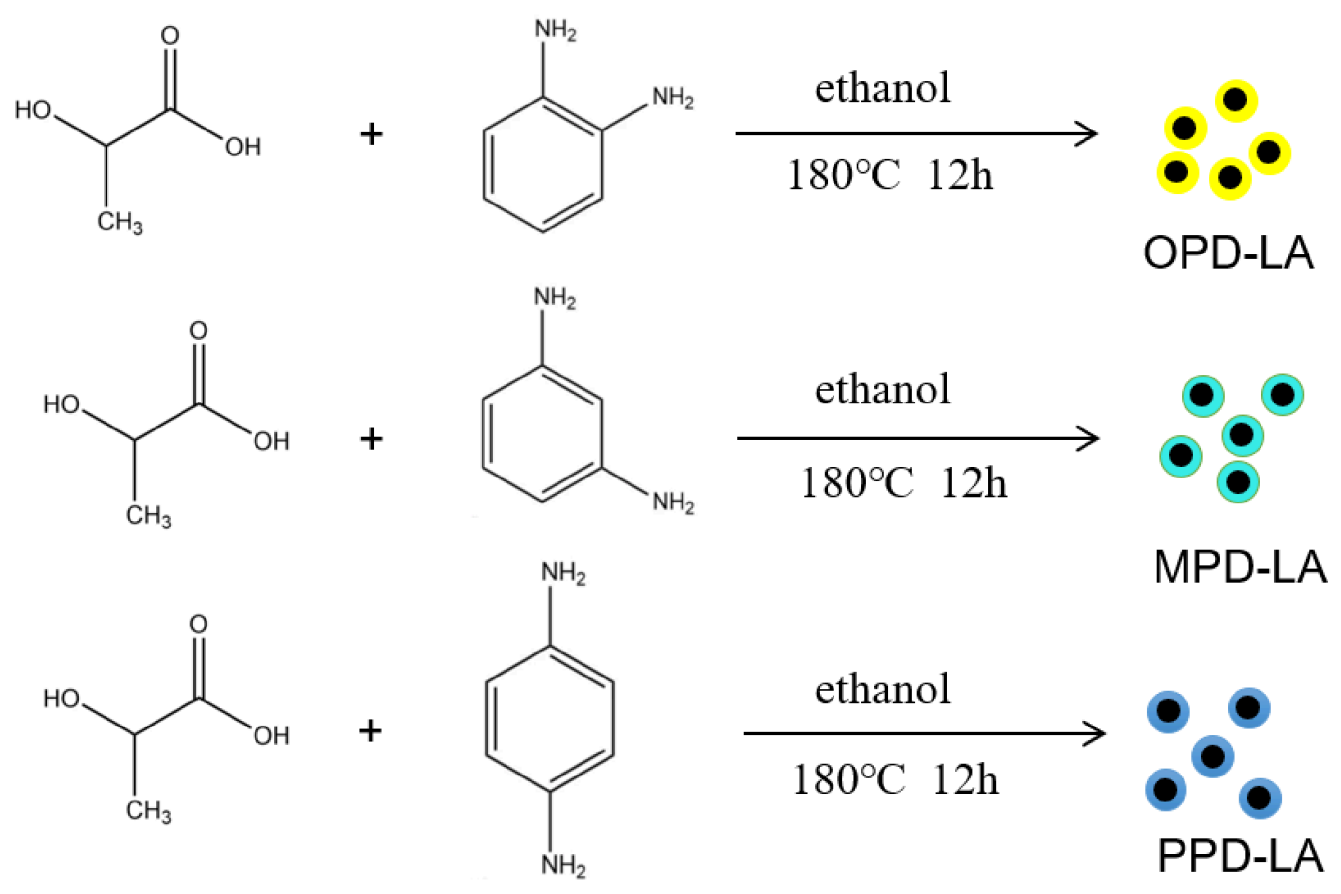
Three different phenylenediamine isomers (oPD, mPD and pPD) were used to prepare lactic acid-based CQDs (designated as OPD-LA, MPD-LA and PPD-LA, respectively).

In this work, N-CQDs were fabricated through a one-pot hydrothermal procedure, as depicted in Scheme 1. The experimental steps are described in detail below: 0.25 g of L-lactic acid and 0.25 g of phenylenediamine isomer were dissolved in 25 mL of ethanol and blended homogeneously. After ultrasonic treatment for 10 min, the mixture was transferred into a Teflon-lined stainless-steel autoclave and heated at 180 °C for 12 h to yield the crude N-CQDs dispersion. The obtained product was filtered through a 0.22 μm microporous membrane to remove large particle impurities, followed by dialysis for 72 h using a dialysis bag with a molecular weight cutoff of 3500 Da. Finally, the sample was dried at 40 °C for subsequent characterization. Three lactic acid-based CQDs derived from o-phenylenediamine, m-phenylenediamine, and p-phenylenediamine were designated as OPD-LA, MPD-LA, and PPD-LA, respectively.

To investigate the effect of L-lactic acid on the surface structure and properties of CQDs, a series of controlled CQDs was prepared through the same procedure mentioned above, only using the three phenylenediamine isomers as both carbon and nitrogen sources without the addition of L-lactic acid. The obtained CQDs were labeled as o-phenylenediamine-based CQDs (OPD), m-phenylenediamine-based CQDs (MPD) and p-phenylenediamine-based CQDs (PPD).

### 2.4. Measurement of Quantum Yields

The relative fluorescence quantum yield (QY) of the as-prepared N-CQDs was measured with quinine sulfate (QS) employed as the reference standard. (Solvent: 0.1 M H_2_SO_4_; Φ_QS_ = 0.54) [30]. To ensure reliable and repeatable data, the absorbance values of N-CQDs and QS solutions at 360 nm were regulated within the range of 0.02–0.1. The fluorescence quantum yield of N-CQDs was calculated using the equation given below.

(1) $$\Phi_{x}=\;\Phi_{\mathrm{QS}}\frac{K_{x}}{K_{\mathrm{QS}}}\frac{\eta_{x}^{2}}{\eta_{\mathrm{QS}}^{2}}$$

In the equation, Φ_QS_ represents the documented quantum yield of quinine sulfate in 0.1 M H_2_SO_4_ (fixed at 0.54). K stands for the slope obtained from the plot of integrated fluorescence intensity versus absorbance for both the sample and reference. η refers to the refractive index of the solvent, which is 1.33 for deionized water.

### 2.5. Effects of pH Value, Ionic Concentration and Time on the Fluorescence Intensity of N-CQDs

The N-CQDs were dispersed in deionized water to prepare a homogeneous solution with a concentration of 0.05 mg/mL. Then, 3 mL of the N-CQDs solution was thoroughly mixed with 3 mL of hydrochloric acid or sodium hydroxide solution of preset pH values. Buffer solutions were not employed, as the coexisting ions in buffers may interact with the surface functional groups of N-CQDs, interfering with their intrinsic fluorescence signals and recognition capability toward Fe^3+^. Therefore, pure hydrochloric acid and sodium hydroxide were used for pH regulation to minimize external ion interference. After adjusting the pH of the solution with HCl or NaOH, the sample was allowed to stand for 10 min to reach pH equilibrium. Subsequently, we rechecked and recorded the exact pH value immediately before photoluminescence measurement. All fluorescence spectra were collected uniformly after this fixed equilibration time.

To evaluate the anti-interference performance, NaCl solutions with concentrations ranging from 0 to 3.0 mol/L were prepared as the interfering ionic system. Afterward, 3 mL of CQDs dispersion was fully blended with 3 mL of NaCl solution and ultrasonicated for 15 min. Fluorescence intensity was then measured at the optimal excitation wavelength.

The as-prepared CQDs dispersion was stored in the dark at ambient temperature. Fluorescence intensity measurements were carried out at the optimal excitation wavelength after storage for 0, 1, 2, 3, 4, 5, and 6 months, respectively.

### 2.6. Fluorescence Detection of Fe^3+^ Ions

FeCl_3_ solutions with different concentrations were prepared by dissolving ferric chloride in deionized water. To investigate the detection scope and sensing responsiveness of N-CQDs toward Fe^3+^ ions, 3 mL of Fe^3+^ solutions (0–200 μM) were mixed thoroughly with 3 mL of aqueous N-CQDs dispersion at ambient temperature. Fluorescence tests were then performed on the mixed solutions. The excitation wavelength for all fluorescence measurements was fixed at 360 nm, and the fluorescence intensity at the corresponding maximum emission peak was recorded.

## 3. Results and Discussion

### 3.1. Morphology and Chemical Composition of CQDs

**Figure 1:**
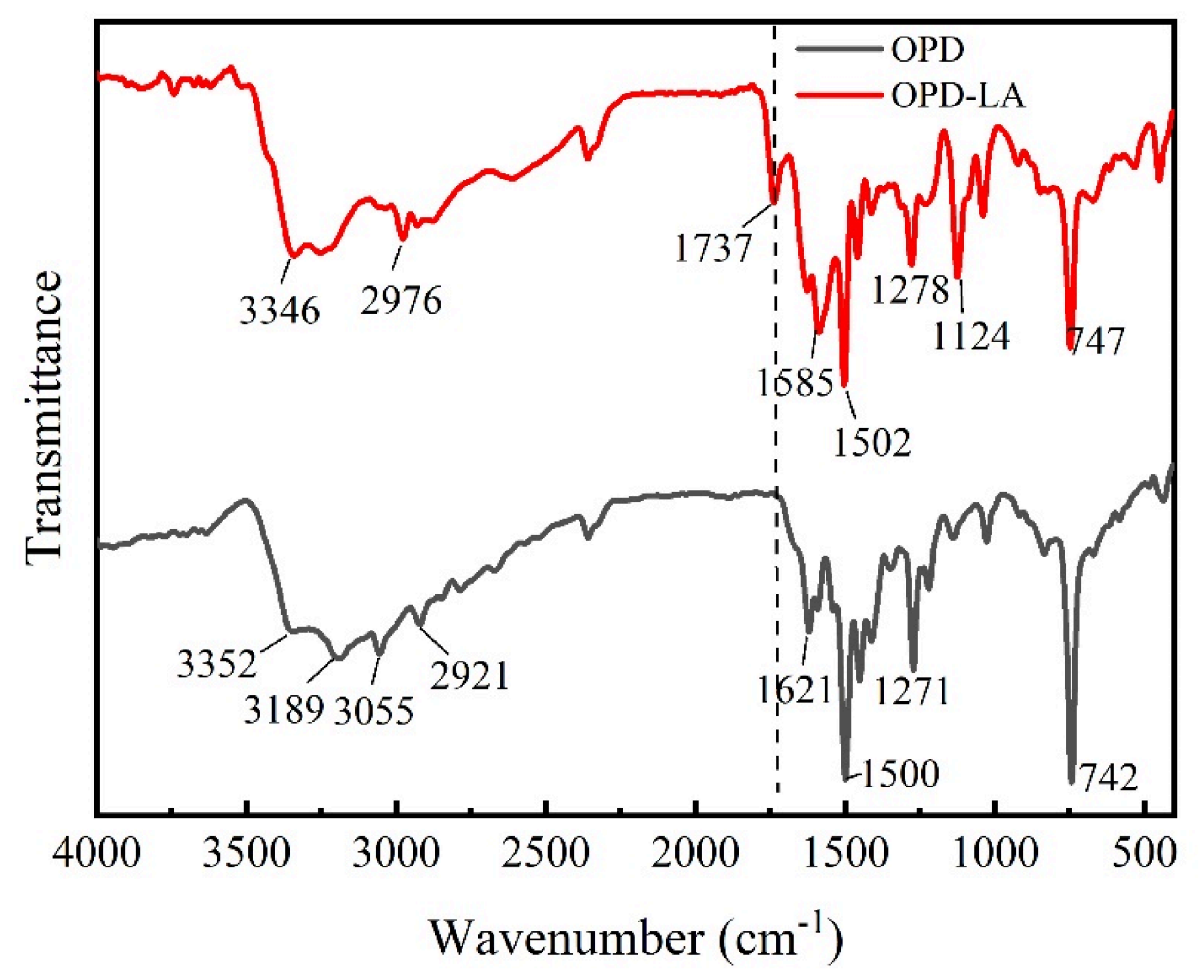
FTIR spectrum of OPD and OPD-LA.

The surface composition, group distribution and surface morphology of CQDs exert a remarkable influence on their properties. First of all, Fourier-transform infrared (FTIR) spectroscopy was utilized to identify the surface functional moieties of the as-prepared CQDs. Figure 1 displays the FTIR spectra of OPD and OPD-LA. Both types of CQDs show characteristic absorption peaks around 3350 cm^−1^, 2950 cm^−1^ and 1500–1600 cm^−1^, which are assigned to the stretching vibrations of N–H/O–H, aliphatic C–H, and aromatic skeleton C=C [31], respectively, confirming the presence of amino groups and aromatic structures in the products, which was distinct from the previous ethylenediamine-doped CQDs.

Compared with OPD, OPD-LA exhibits two new absorption peaks at 1740 cm^−1^ and 1685 cm^−1^, which can be attributed to the C=O stretching vibration of carboxyl groups and amide groups, respectively. This result verifies the successful introduction of lactic acid structural units. The carboxyl groups of lactic acid reacted with one or two amino groups of o-phenylenediamine, thereby forming products containing amide bonds and side-chain hydroxyl groups.

Similarly, the FTIR spectra of a series of carbon quantum dots synthesized from m-phenylenediamine, p-phenylenediamine and lactic acid (Figures S1 and S2) are similar to those of OPD-LA. MPD-LA and PPD-LA may also contain N–H, O–H, carbonyl and amide groups on their surfaces, while the content of carbonyl groups is relatively lower.

**Figure 2:**
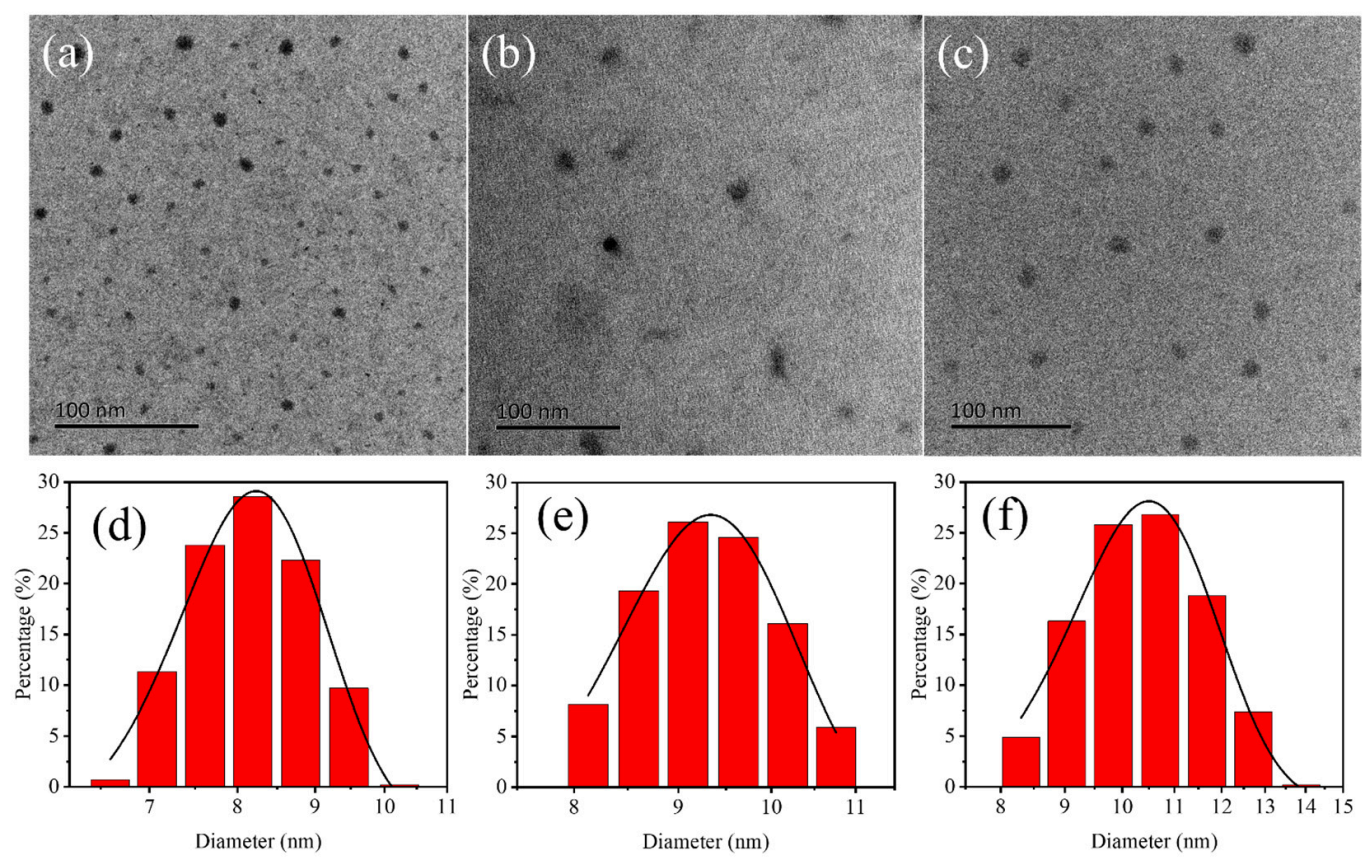
TEM image of (**a**) OPD-LA, (**b**) MPD-LA, (**c**) PPD-LA and particle size histogram of (**d**) OPD-LA, (**e**) MPD-LA, (**f**) PPD-LA.

The size and surface morphology of CQDs are crucial to the distribution of surface functional groups, surface properties, and their dispersion performance in polymer matrices. On the basis of clarifying the types of surface groups by FTIR spectroscopy, transmission electron microscopy (TEM) combined with particle size analysis was used to investigate the morphological characteristics and particle size distribution of three fluorescent N-CQDs, namely OPD-LA, MPD-LA and PPD-LA. As shown in Figure 2, all three samples present uniformly dispersed nanoparticle morphology. The average particle size of OPD-LA is approximately 8.2 nm, MPD-LA is about 9.3 nm, and PPD-LA reaches 10.5 nm. OPD-LA possesses the smallest average particle size. This may be attributed to the fact that the two amino groups (–NH_2_) in o-phenylenediamine are spatially closest to each other, which facilitates cyclization, condensation and carbonization reactions, thereby accelerating the nucleation rate, increasing the number of nuclei and yielding smaller particle sizes. The average particle size of MPD-LA is larger than that of OPD-LA. The two amino groups in m-phenylenediamine are relatively far apart, which is unfavorable for rapid condensation and nucleation. Consequently, the nucleation process proceeds more slowly, resulting in a larger particle size. PPD-LA exhibits the largest particle size among the three samples. For p-phenylenediamine, the two amino groups are located at the para-position with high molecular symmetry. The reaction system tends to form long-chain polymers rather than rapid nucleation, leading to fewer crystal nuclei and ultimately the largest particle size.

**Figure 3:**
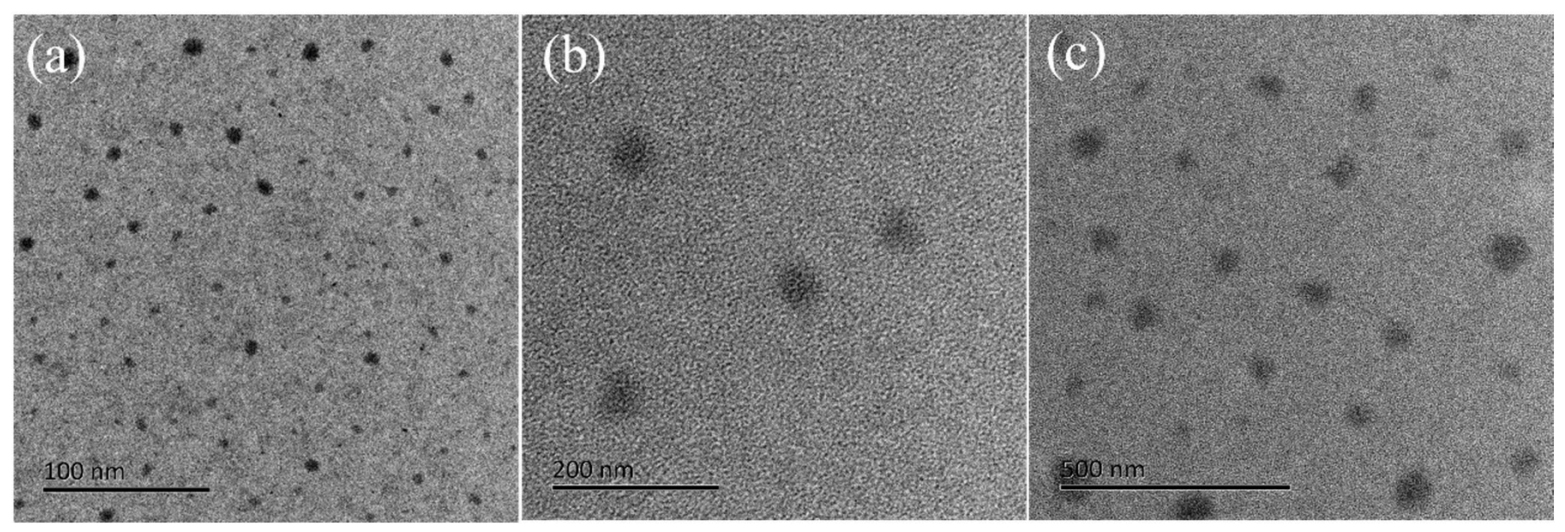
TEM image of (**a**) OPD-LA 12 h, (**b**) OPD-LA 24 h and (**c**) OPD-LA 48 h.

To explore the evolution law of N-CQD particle size, N-CQDs were prepared under different reaction durations. The TEM results show that the particle size of N-CQDs increases with the extension of reaction time (Figure 3). After nucleation, small molecules or oligomers in the solution can further attach to the surface of crystal nuclei and undergo continuous dehydration and carbonization, forming highly cross-linked carbon frameworks with graphite-like or diamond-like structures. The formation of new surface layers and the continuous growth of carbon nuclei jointly promote the increase in CQD particle size. Therefore, longer reaction time leads to a larger size of the as-obtained N-CQDs [32,33].

**Figure 4:**
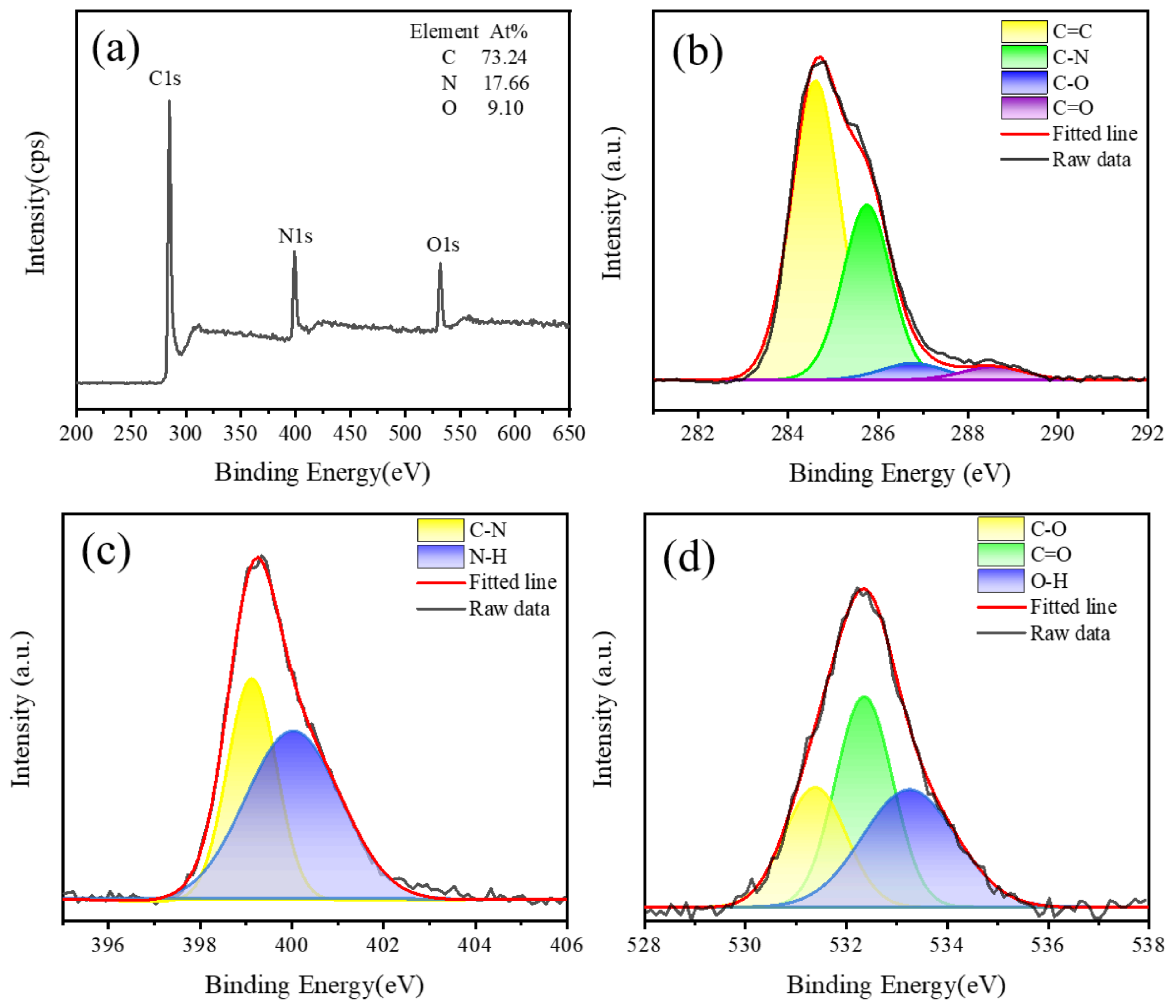
XPS spectra of OPD-LA CQDs (**a**) and high-resolution XPS spectra of (**b**) C1s, (**c**) N1s, and (**d**) O1s.

The chemical valence states and distribution of surface groups of N-CQDs are critical to their comprehensive properties. On the basis of FTIR analysis of surface functional group types, X-ray photoelectron spectroscopy (XPS) was performed on OPD-LA to further investigate the surface chemical composition and group distribution of N-CQDs. As shown in Figure 4, OPD-LA is rich in carbon, nitrogen and oxygen elements. The characteristic peaks at 284.8 eV, 399.5 eV and 532.3 eV correspond to C1s, N1s and O1s orbits, with normalized atomic percentages of 73.24%, 17.66% and 9.10%, respectively [34,35,36]. The high-resolution C1s spectrum illustrated in Figure 4b can be fitted into four distinct peaks located at 284.6 eV, 285.7 eV, 286.8 eV, and 288.6 eV, which are ascribed sequentially to C=C, C–N, C–O, and C=O bonds. The high-resolution N1s spectrum in Figure 4c presents two typical peaks at 399.1 eV and 400.1 eV, verifying the presence of C–N and N–H groups. The high-resolution O1s spectrum shown in Figure 4d exhibits three fitted peaks at 531.4 eV, 532.4 eV, and 533.2 eV, associated with C–O, C=O, and O–H bonds, respectively. On the basis of FTIR and XPS analyses, it can be concluded that the as-synthesized N-CQDs contain a nitrogen-doped conjugated aromatic carbon framework, and their surfaces are decorated with diverse hydrophilic functional groups, including –COOH/–COO^−^ amide linkage –C=O(NH)–, C=N, –OH, and –NH–. These abundant polar groups impart outstanding water solubility to the N-CQDs. Furthermore, the lone-pair electrons on O and N atoms can establish p-π conjugation with the extended π-conjugated system of the carbon core, which endows the N-CQDs with intense fluorescence emission.

### 3.2. UV–Vis Absorption and Fluorescence Spectra of N-CQDs

**Figure 5:**
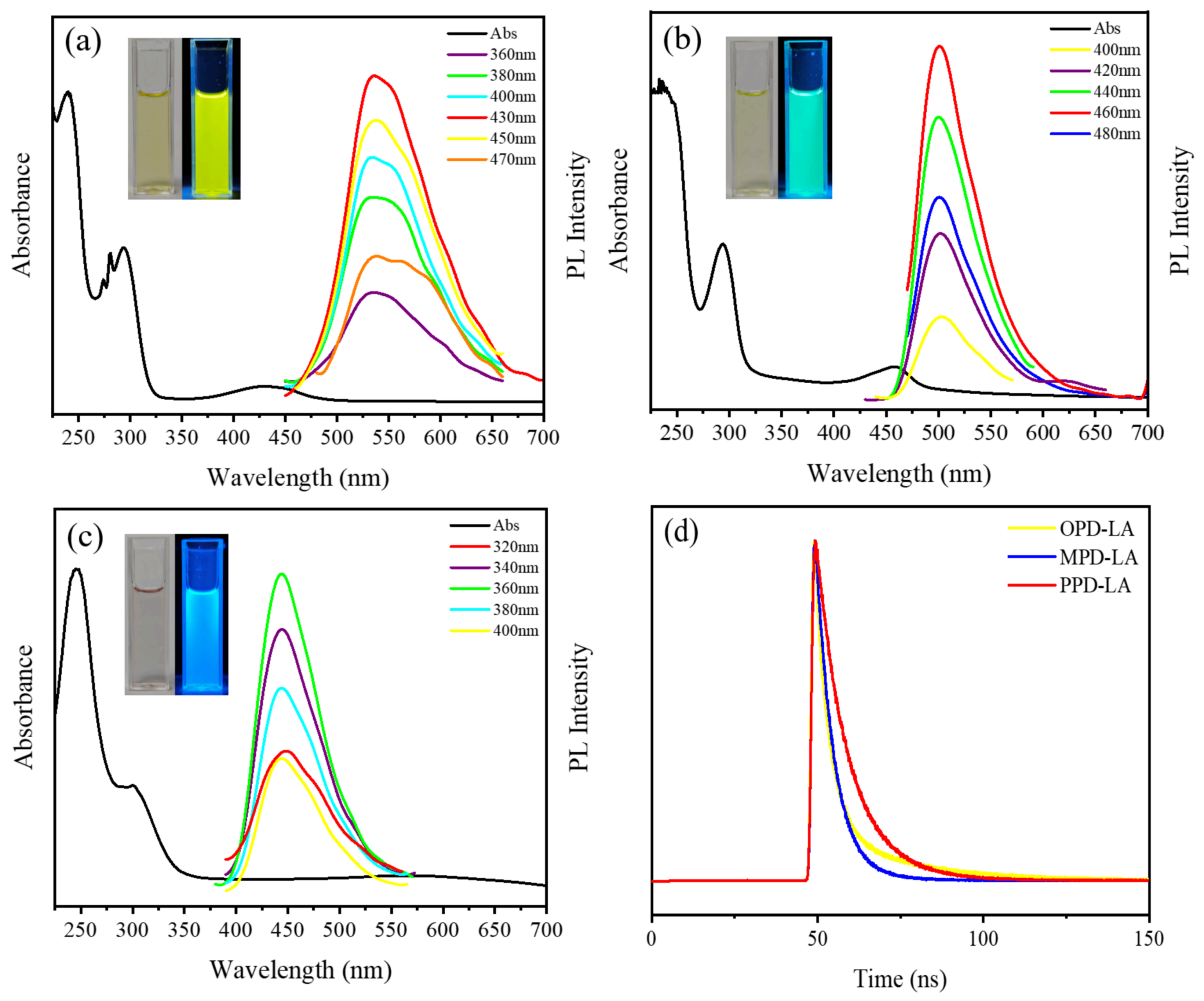
Absorption spectra and PL emission spectra of (**a**) OPD-LA, (**b**) MPD-LA, and (**c**) PPD-LA under excitation with different wavelengths of light. (**d**) PL decay spectra of three selected samples.

FTIR and XPS results indicate that N-CQDs possess not only a nitrogen-doped conjugated aromatic carbon core but also abundant hydrophilic functional groups on the surface, which endow N-CQDs with distinctive fluorescence properties and photoluminescence (PL) behavior. Figure 5 presents the UV-vis absorption spectra, photoluminescence (PL) emission spectra, and PL decay curves of OPD-LA, MPD-LA, and PPD-LA. All three samples display distinct absorption features in both the ultraviolet and visible light regions. The strong absorption peak near 250 nm is a typical characteristic of CQDs, which originates from the π-π* electronic transition of sp^2^-hybridized carbon in the carbon core (conjugated C=C system), directly verifying the existence of a graphite-like carbon core in N-CQDs. The minor absorption shoulder at around 300 nm is ascribed to the n-π* electron transition of surface functional groups, including –COOH, –OH, and C=O. This indicates that the CQD surface is abundant in polar oxygen- and nitrogen-containing moieties, which structurally provide the CQDs with superior water dispersibility [37]. As shown in Figure S3 in the Supplementary materials, the optimal excitation wavelengths of PPD-LA, OPD-LA and MPD-LA are 360 nm, 430 nm and 460 nm, respectively, showing a gradual red shift and suggesting different surface states among the three CQD samples [38]. The maximum emission peaks of PPD-LA, OPD-LA and MPD-LA are separately located at 450 nm, 550 nm and 505 nm. In addition, unlike the previous excitation-dependent blue fluorescence, the as-prepared CQDs showed excitation-independent multicolor fluorescence (yellow, turquoise, blue). The well-defined emission peak shape and narrow full width at half maximum (FWHM) indicate uniform luminescence centers and stable energy level structures of this series of CQDs.

To explore the luminescence lifetime characteristics and charge recombination mechanism of the as-prepared N-CQDs, as well as to clarify the contribution of different energy states in the luminescence process, time-resolved photoluminescence decay curves were measured for each sample. The results are presented in Figure S4 and summarized in Table 1. The decay data were well fitted by a biexponential model, which consists of a short-lived component τ_1_ and a long-lived component τ_2_, corresponding to the charge recombination processes of the carbon core state and surface state, respectively [39,40]. The short-lived component (τ_1_) dominates in all three samples (A_1_ > 82%), indicating that the intrinsic transition originating from the carbon core serves as the major luminescence channel. In contrast, the long-lived component (τ_2_) exhibits obvious differences in both amplitude (A_2_) and lifetime value, which are closely related to the surface structure, defect density and charge recombination kinetics of the materials. OPD-LA shows the highest proportion of the long-lived component (A_2_ = 0.17834) and the longest average fluorescence lifetime (τ_avg_ = 14.49 ns). By comparison, MPD-LA possesses the lowest amplitude of the long-lived component (A_2_ = 0.00629) and the shortest average lifetime (τ_avg_ = 6.44 ns). Both A_2_ and the average lifetime of PPD-LA are at a moderate level, reflecting a relatively balanced contribution of the carbon core state and surface state to luminescence. The variations in the relative proportion and magnitude of the two lifetime components demonstrate that surface structure and defect distribution play a dominant regulatory role in the photoluminescence performance of the prepared CQDs.

To verify the rationality of photoluminescence measurement conditions, eliminate the interference of the inner filter effect, quantitatively evaluate the fluorescence efficiency of each sample, and explore the regulation effect of lactic acid introduction on the fluorescence performance of N-CQDs, the relationship between photoluminescence intensity and absorbance of quinine sulfate, OPD-LA, MPD-LA and PPD-LA was measured, and the fluorescence quantum yields were calculated accordingly. Figure S5 presents the linear fitting curves of photoluminescence intensity versus absorbance for quinine sulfate, OPD-LA, MPD-LA and PPD-LA. All samples exhibit good linear correlation within the absorbance range of 0.02–0.10, which verifies the rationality of the experimental conditions and confirms that no obvious inner filter effect exists. The slopes of the fitting curves and the calculated quantum yields (QY) are summarized in Table 2. The results show that the fluorescence quantum yields of OPD-LA, MPD-LA and PPD-LA are 8.48%, 9.59% and 2.20%, respectively. The slopes and quantum yields follow the order: MPD-LA (4.416 × 10^7^, 9.59%) > OPD-LA (3.903 × 10^7^, 8.48%) > PPD-LA (1.016 × 10^7^, 2.20%). Meanwhile, the fluorescence quantum yields of pure phenylenediamine-based CQDs (OPD, MPD and PPD) were also tested, which are 2.29%, 3.75% and 1.36%, respectively. By comparison, the quantum yields of OPD-LA, MPD-LA and PPD-LA synthesized with the introduction of lactic acid are all significantly higher than those of the corresponding pure phenylenediamine counterparts. It is demonstrated that the introduction of lactic acid exerts a prominent positive regulatory effect on improving fluorescence quantum yield. This is mainly attributed to the fact that pure phenylenediamines are small aromatic amine compounds with inherently low fluorescence efficiency, which originates largely from non-radiative transition losses at the molecular level. Strong intermolecular π-π stacking and hydrogen bonding interactions easily cause molecular aggregation in solution, and the excited-state energy is mostly dissipated through non-radiative pathways, resulting in obvious fluorescence quenching. Furthermore, the free amino groups in the monomers are susceptible to solvent environments and protonation/deprotonation effects, tending to form unstable excited-state intermediates and further reducing the probability of radiative recombination. The quantum yield was regulated by phenylenediamine isomers, which was a new regulation strategy different from our previous work.

Under hydrothermal conditions, lactic acid reacts with phenylenediamine through dehydration, polycondensation and carbonization to in situ form N-CQDs, which suppress non-radiative losses from the perspective of structure and electronic states and thereby improve the quantum yield. Lactic acid undergoes dehydration, decarboxylation and carbonization to form a sp^2^-hybridized conjugated carbon core, while phenylenediamine is covalently incorporated into the carbon skeleton via C–N and C–C bonds, extending the small-molecule conjugated system into a rigid large conjugated carbon network. The rigid structure effectively restricts intramolecular vibration and rotation, reduces non-radiative energy dissipation, and the expanded conjugated system facilitates the stable existence of charge carriers and their recombination in the form of fluorescence [41]. Moreover, the amino groups of phenylenediamine react with the carboxyl and hydroxyl groups of lactic acid through amidation and cyclization under hydrothermal conditions. Nitrogen atoms are stably doped into the carbon skeleton in the forms of C–N and N–H groups. These N-doping sites act as radiative recombination centers, optimize the electronic structure and improve the utilization efficiency of excited states [42]. Meanwhile, lactic acid introduces abundant polar groups such as hydroxyl and carboxyl groups into N-CQDs, remarkably enhancing the aqueous dispersion of N-CQDs. This avoids concentration quenching and aggregation-caused quenching, stabilizes the surface electronic states, and reduces non-radiative quenching pathways induced by structural defects.

### 3.3. Fluorescence Stability of N-CQDs

**Figure 6:**
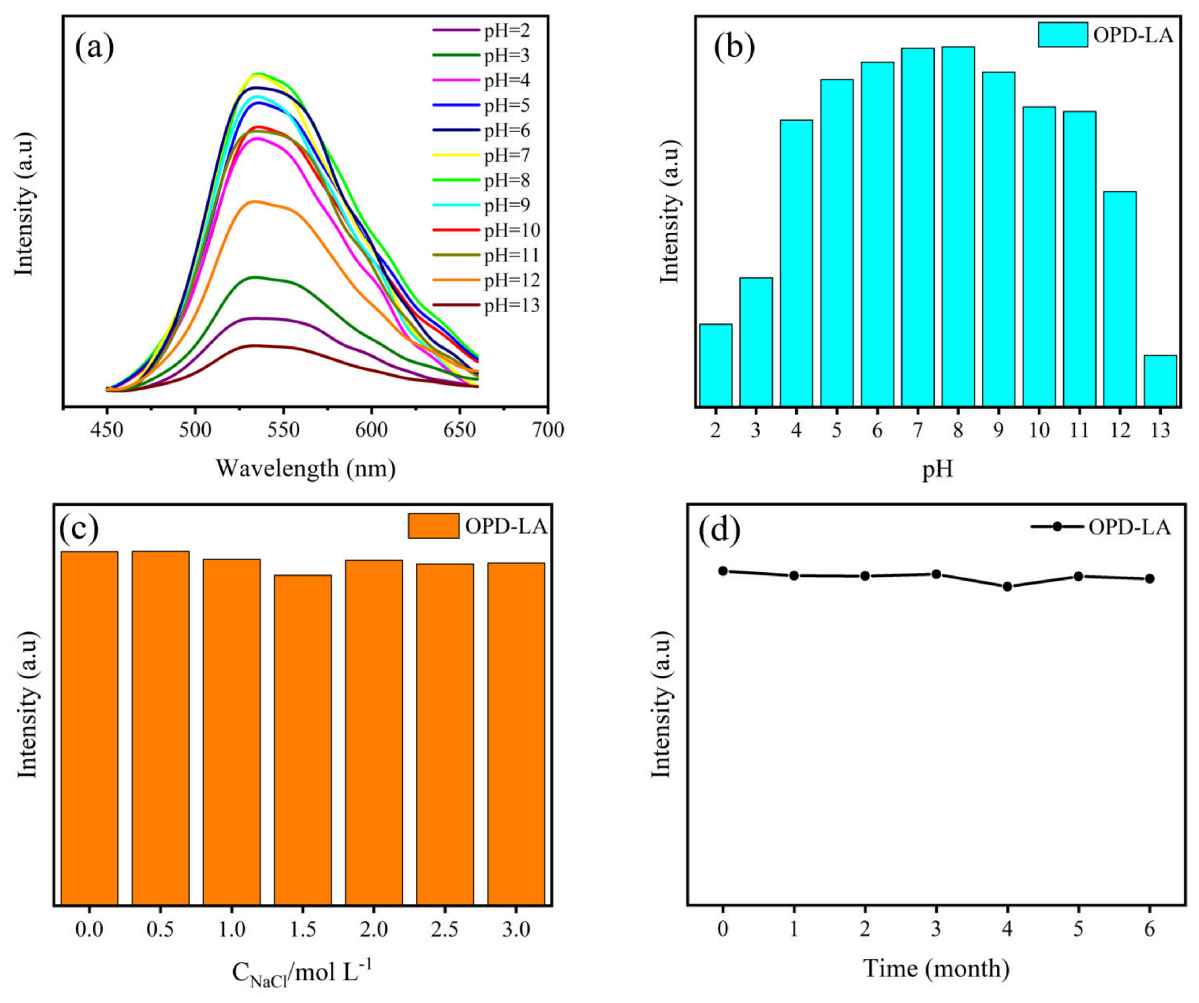
The fluorescence spectra and fluorescence intensity of OPD-LA at various pH values (**a**) and (**b**), respectively; (**c**) shows the fluorescence intensity of OPD-LA at different NaCl concentrations; (**d**) represents the variation in fluorescence intensity of the same OPD-LA solution at room temperature over 6 months.

Owing to their remarkable sensitivity, low cytotoxicity, and tunable luminescence characteristics, N-CQDs show considerable application promise in light-emitting diodes, solar cells, bioimaging, cell imaging, and the detection of food additives and pharmaceutical residues. The stability of fluorescence features is critical for their real-world applications. Therefore, the influences of pH value, ionic concentration, and storage duration on the fluorescence behavior of the synthesized N-CQDs were systematically explored in this part. The fluorescence intensity of OPD-LA under different pH conditions is shown in Figure 6a. OPD-LA maintains high and stable fluorescence intensity within the pH range of 4–11 (Figure 6b). Nevertheless, strongly acidic (pH < 4) or strongly alkaline (pH > 11) environments lead to a decrease in fluorescence intensity, and the fluorescence quenching becomes more severe as the pH approaches extreme values. This phenomenon can be attributed to abundant surface functional groups of OPD-LA, including carboxyl, hydroxyl, amide and amino groups [43]. Under extreme acidic and alkaline conditions, the protonation–deprotonation process occurs on the surface of OPD-LA, causing obvious changes in surface charge and further triggering aggregation-induced fluorescence quenching. The influence of ionic strength on OPD-LA is presented in Figure 6c. When the NaCl concentration varied from 0 to 3 mol·L^−1^, the fluorescence intensity of OPD-LA remained nearly unchanged, indicating excellent fluorescence stability of OPD-LA even under high ionic strength. Furthermore, the CQD solution was stored at room temperature for six months, and the fluorescence intensity at 540 nm was monitored every month to evaluate its long-term stability. As shown in Figure 6d, no obvious attenuation of fluorescence intensity was observed within six months, demonstrating the outstanding storage stability of the OPD-LA solution. The corresponding results of MPD-LA and PPD-LA are displayed in Figure S6 and Figure S7, respectively. Their fluorescence intensity only slightly decreased within six months, further confirming the superior fluorescence stability of the prepared N-CQDs.

### 3.4. Detection Analysis of N-CQDs Toward Fe^3+^

**Figure 7:**
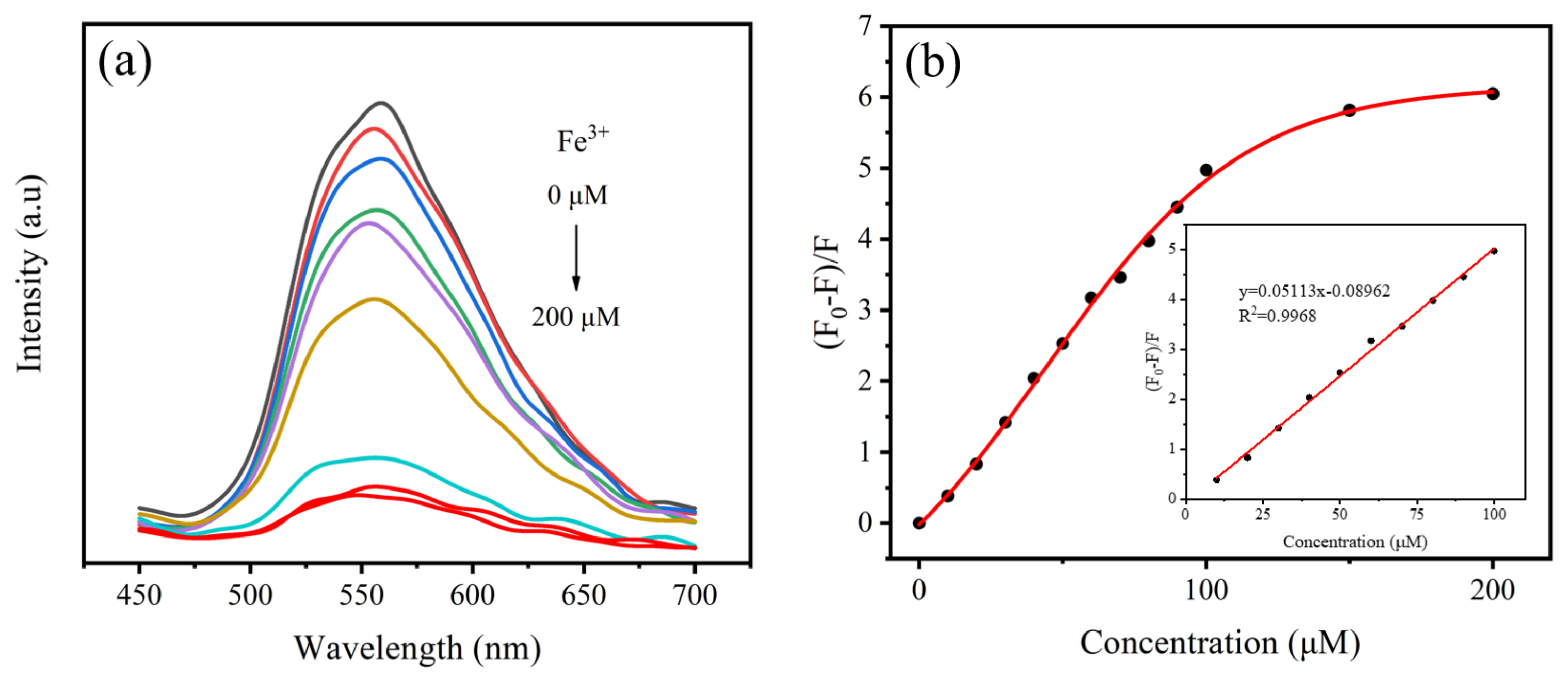
(**a**) Fluorescence spectra of N-CQDs dispersions with gradient Fe^3+^ concentrations ranging from 0 to 200 μM; (**b**) fluorescence intensity ratio (*F*_0_/*F*) of OPD-LA as a function of Fe^3+^ concentration, where *F*_0_ and *F* correspond to the fluorescence intensity of OPD-LA in the absence and presence of Fe^3+^ ions, respectively. The inset depicts the good linear correlation between the *F*_0_/*F* value and Fe^3+^ content within the concentration range of 10–100 μM.

It can be concluded from the above analysis that the prepared N-CQDs possess the advantages of high sensitivity, excellent stability and tunable fluorescence, and can be potentially used as fluorescent probes for the detection of metal ions and pesticide residues. In view of the outstanding fluorescence stability of OPD-LA, it was selected as the fluorescent probe for the detection and analysis of Fe^3+^. Based on the fluorescence quenching effect of Fe^3+^ toward N-CQDs, the Stern–Volmer equation was adopted to establish a quantitative analytical method for the rapid determination of Fe^3+^ concentration in water. In the experiment, a series of Fe^3+^ solutions with concentrations ranging from 0 to 200 μM were mixed with an equal volume of N-CQDs solution. After sufficient ultrasonic dispersion, the fluorescence emission spectra were recorded (Figure 7a). The Stern–Volmer equation is a classical quantitative model for fluorescence quenching analysis, which correlates (*F*_0_ − *F*)/*F* with the quencher concentration [44], and its expression is given as:

(2) $${(F}_{0}-F)/F=KSV[C]$$

The limit of detection (*LOD*) of the sample was calculated using the following formula:

(3) $$LOD=\frac{3\sigma }{KSV}$$

*σ* refers to the standard deviation of fluorescence intensity of blank samples, measured no less than ten times in parallel.

To evaluate the ion selectivity of the OPD-LA fluorescent probe, equal volumes of 3 mM aqueous solutions containing different metal cations (Na^+^, K^+^, Ca^2+^, Mg^2+^, Mn^2+^, Zn^2+^, Cu^2+^, Nb^5+^, Fe^3+^) were individually mixed with N-CQD dispersion (2.0 μg mL^−1^). After sufficient blending, the fluorescence spectra of all mixtures were collected under an excitation wavelength of 360 nm.

Ideally, a linear correlation exists between them. The slope *KSV* of the fitting curve represents the fluorescence quenching constant with the unit of L·mol^−1^. It reflects the susceptibility of fluorescent substances to quenching agents, and a larger value indicates stronger quenching capacity. Nevertheless, the experimental data fail to exhibit an ideal linear fitting trend (Figure 7b), which is primarily ascribed to the rich functional groups anchored on the N-CQD surface. As the quencher concentration increases, electron transfer can occur simultaneously at multiple surface-active sites, leading to the coexistence of multiple quenching constants and eventually resulting in fluctuations in the quenching behavior. The specific binding interactions between Fe^3+^ and the carboxyl, amino, and hydroxyl groups on N-CQDs are the primary cause of fluorescence quenching. Within the concentration range of 10–100 μM, the experimental data fit well with the Stern–Volmer equation and exhibit an excellent linear relationship. The linear regression equation is: (*F*_0_ − *F*)/*F* = 0.05113[C] − 0.08962 (R^2^ = 0.9968). The corresponding result is shown in the inset of Figure 7b. According to the three-standard-deviation calculation criterion for blank samples, the limit of detection (*LOD*) of the synthesized N-CQDs for Fe^3+^ was determined to be 1.49 μM. Compared with previously reported carbon quantum dots, the as-prepared N-CQDs in this work possess a wider detection range and favorable sensing sensitivity (Table 3).

**Figure 8:**
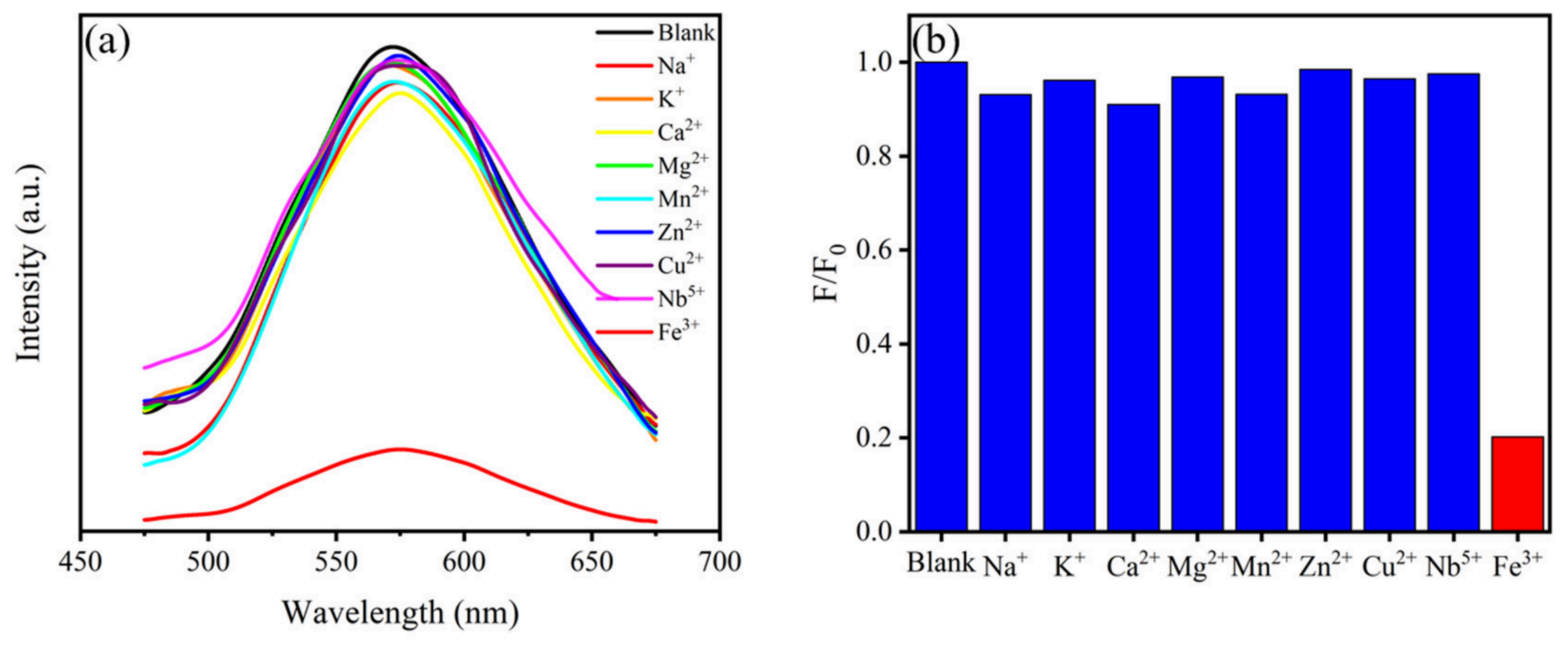
Fluorescence spectra (**a**) and normalized histogram (**b**) showing the effects of different metal ions on the fluorescence intensity of OPD-LA.

As depicted in the fluorescence spectra (Figure 8a), the characteristic emission peak of OPD-LA at approximately 580 nm was remarkably quenched only after the addition of Fe^3+^. Other metal ions, including Na^+^, K^+^, Ca^2+^, Mg^2+^, Mn^2+^, Zn^2+^, Cu^2+^ and Nb^5+^, exerted nearly no influence on the fluorescence intensity of the system. The normalized histogram in Figure 8b further quantifies this trend. The *F*/*F*_0_ values of the probe remained above 0.9 in the presence of all interfering ions, while the value dropped sharply to around 0.2 when Fe^3+^ was added, corresponding to a fluorescence quenching efficiency of 80%. The above experimental results demonstrate that the probe exhibits favorable preliminary selectivity under laboratory conditions, showing potential for detection in actual water samples.

## 4. Conclusions

In this work, a series of lactic acid-based CQDs were fabricated via a hydrothermal method using three phenylenediamine isomers (oPD, mPD, pPD) as nitrogen sources and L-lactic acid as both a carbon source and modifier. Their microstructure, surface groups, fluorescence properties and Fe^3+^ detection application were systematically investigated. In terms of morphology and composition, the as-prepared OPD-LA, MPD-LA and PPD-LA are all uniformly dispersed nanoparticles with average particle sizes of 8.2 nm, 9.3 nm and 10.5 nm, respectively. The particle size is affected by the spatial position of amino groups in phenylenediamine and the reaction time. Using lactic acid as the carbon source and phenylenediamine as the nitrogen source, the obtained N-CQDs are endowed with surface functional groups including –OH, –NH, C=C, carbonyl and amide groups. The N-CQDs possess nitrogen-doped conjugated carbon cores and abundant hydrophilic surface groups, thereby exhibiting good water solubility and favorable fluorescence properties. In terms of optical properties, all three N-CQDs show typical UV-vis absorption characteristics, and their optimal excitation wavelengths present a red-shift trend with excitation-independent stable photoluminescence. The fluorescence lifetime is dominated by the short-lived component. The fluorescence quantum yield follows the order: MPD-LA (9.59%) > OPD-LA (8.48%) > PPD-LA (2.20%), all of which are higher than those of the corresponding pure phenylenediamine-based CQDs. It indicates that the introduction of lactic acid not only modulates the surface groups of CQDs but also positively regulates their fluorescence performance. In terms of fluorescence stability, OPD-LA, MPD-LA and PPD-LA maintain stable fluorescence performance within the pH range of 4–11 and NaCl concentrations of 0–3 mol·L^−1^. No obvious decrease in fluorescence intensity is observed after six months of storage at room temperature, which provides a reliable guarantee for practical application. In the application of Fe^3+^ detection using OPD-LA as a fluorescent probe, a good linear relationship is achieved in the concentration range of 10–100 μM with R^2^ = 0.9968, and the limit of detection is 1.49 μM. Compared with the reported CQDs in the literature, the prepared sample exhibits a wider detection range and satisfactory sensitivity, enabling rapid and accurate detection of Fe^3+^ in water. Meanwhile, the OPD-LA probe presents favorable preliminary selectivity under laboratory test conditions.

In summary, high-performance lactic acid-based CQDs were successfully prepared in this work. The structure–activity relationship was clarified, and the positive regulatory effect of lactic acid was verified. Meanwhile, their application potential in Fe^3+^ sensing was developed, which provides a theoretical basis and experimental reference for related research on bio-based carbon quantum dots.

## Figures and Tables

**Table 1:** Photoluminescent lifetimes of OPD-LA, MPD-LA, and PPD-LA in ethanol solutions.

	A_1_	τ_1_ [ns]	A_2_	τ_2_ [ns]	τ_avg_ [ns]
OPD-LA	0.83649	4.06173	0.17834	23.08726	14.49
MPD-LA	1.02105	5.51812	0.00629	32.1454	6.44
PPD-LA	0.97579	9.48313	0.0453	19.39828	10.34

**Table 2:** Photoluminescence quantum yields of quinine sulfate, OPD, MPD, PPD, OPD-LA, MPD-LA, and PPD-LA.

	Quinine Sulfate	OPD	MPD	PPD	OPD-LA	MPD-LA	PPD-LA
Slope	2.623 × 10^8^	1.055 × 10^7^	1.725 × 10^7^	7.651 × 10^6^	3.903 × 10^7^	4.416 × 10^7^	1.016 × 10^7^
QY	54%	2.29%	3.75%	1.36%	8.48%	9.59%	2.2%

**Table 3:** Comparison of CQDs for the determination of Fe^3+^ ions.

Materials	Method	Linear Range (µM)	Limit of Detection (µM)	Reference
Jojoba leaves	Hydrothermal	0.016–0.37	0.018	[45]
Leaves of Peperomia tetraphylla	Hydrothermal	10–100	5.36	[46]
Plastic bags	Hydrothermal	0–100	9.50	[47]
Black plum	Hydrothermal	0–80	0.13	[48]
Tagetes patula flower	Hydrothermal	0–4	0.32	[49]
Biomass waste	Pyrolysis/Microwave	0–50	2.5	[50]
Lactic acid and phenylenediamine	Hydrothermal	10–100	1.49	This work

## Data Availability

The original contributions presented in this study are included in the article/Supplementary Material. Further inquiries can be directed to the corresponding author.

## Supplementary Materials

The following supporting information can be downloaded at: https://www.mdpi.com/article/10.3390/ma19122481/s1, Figure S1. FT-IR spectra of MPD, and MPD-LA. Figure S2. FT-IR spectra of PPD, and PPD-LA. Figure S3. Fluorescence excitation spectrum of OPD-LA (a), MPD-LA (b), and PPD-LA (c). Figure S4. PL decay spectra and fitting curves of OPD-LA (a), MPD-LA (b), and PPD-LA (c). Figure S5. Plots of integrated PL intensity of OPD-LA, MPD-LA, and PPD-LA and quinine sulfate (referenced dye) as a function of optical absorbance at 365 nm and relevant data. Figure S6. The fluorescence spectra and fluorescence intensity of MPD-LA at various pH values, respectively; The fluorescence intensity of MPD-LA at different NaCl concentrations; The variation in fluorescence intensity of the same MPD-LA solution at room temperature over 6 months. Figure S7. The fluorescence spectra and fluorescence intensity of PPD-LA at various pH values, respectively; The fluorescence intensity of PPD-LA at different NaCl concentrations; The variation in fluorescence intensity of the same PPD-LA solution at room temperature over 6 months.
