# Supplementary material for: L-Lactic Acid-Based N-Doped Carbon Quantum Dots with Phenylenediamine Isomers as a Nitrogen Source for the Highly Sensitive Detection of Fe3+ Ions
Source: Materials (Basel). 2026 Jun 10;19(12):2481. doi: 10.3390/ma19122481 (PMC13302333; doi:10.3390/ma19122481)
Supplement: Supplementary file 1 [file materials-19-02481-s001.zip › materials-4345505-supplementary.pdf]

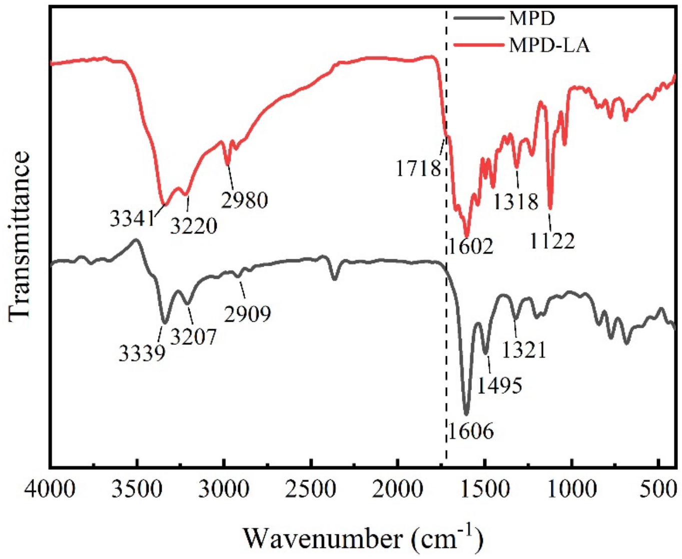

Figure S1. FTIR spectra of MPD and MPD-LA

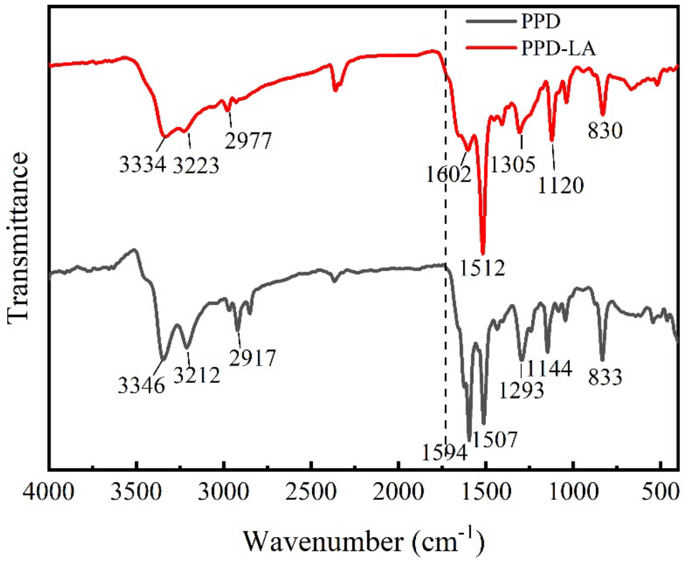

Figure S2. FTIR spectra of PPD and PPD-LA

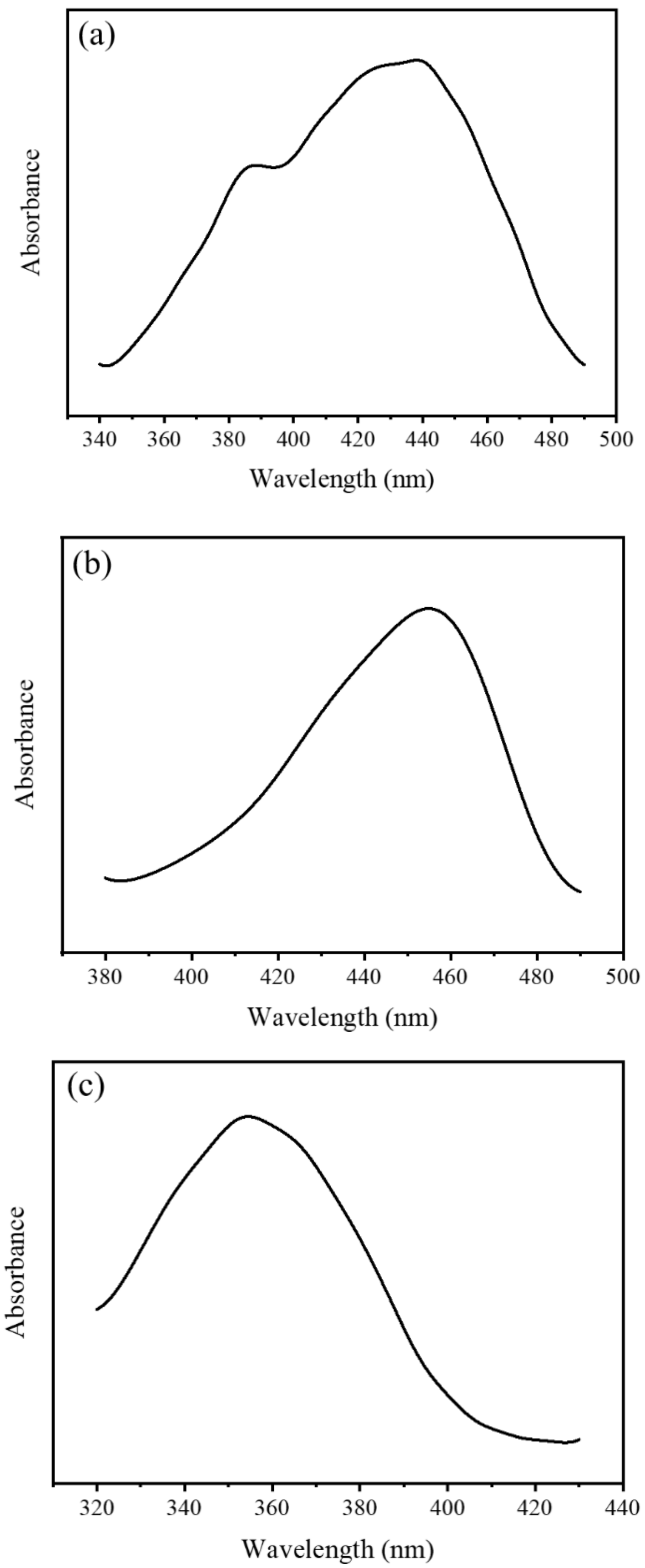

Figure S3. Fluorescence excitation spectrum of OPD-LA (a), MPD-LA (b), and PPD-LA (c).

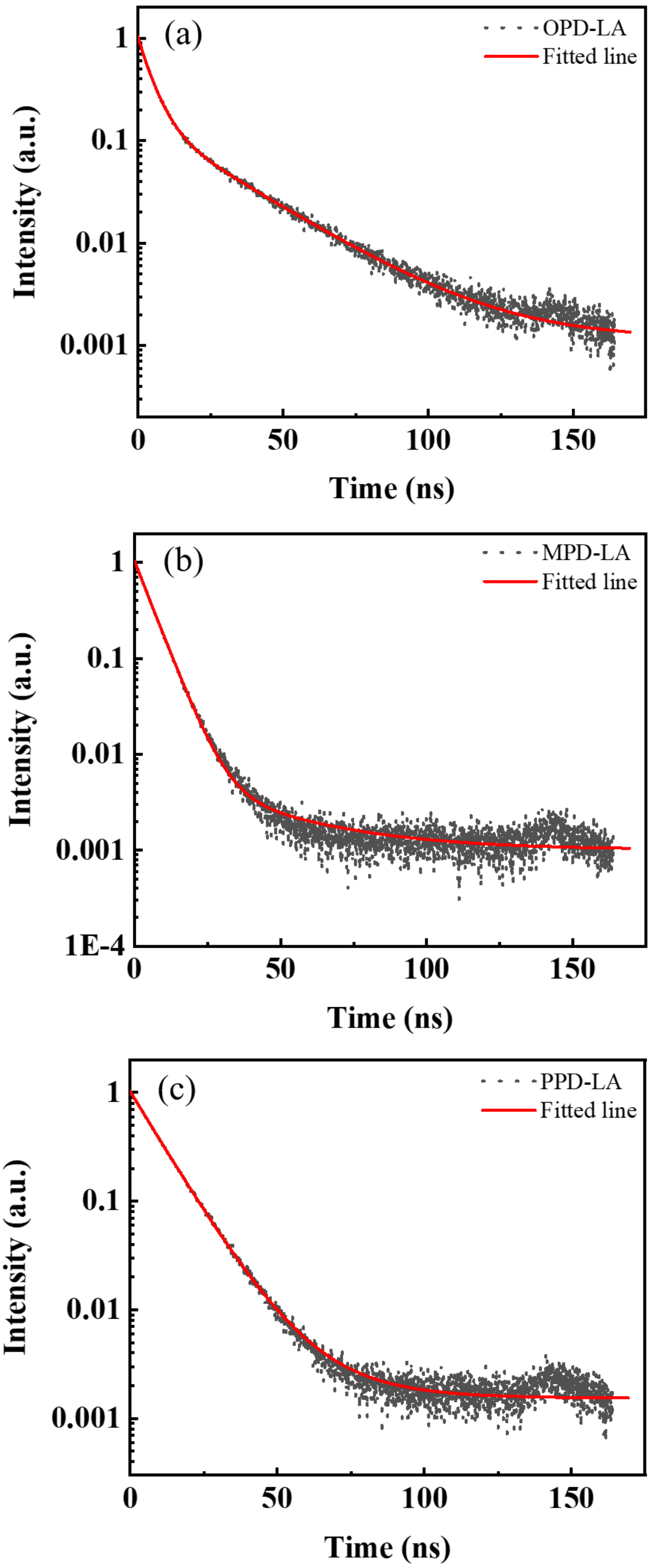

Figure S4. PL decay spectra and fitting curves of OPD-LA (a), MPD-LA (b), and PPD-LA (c).

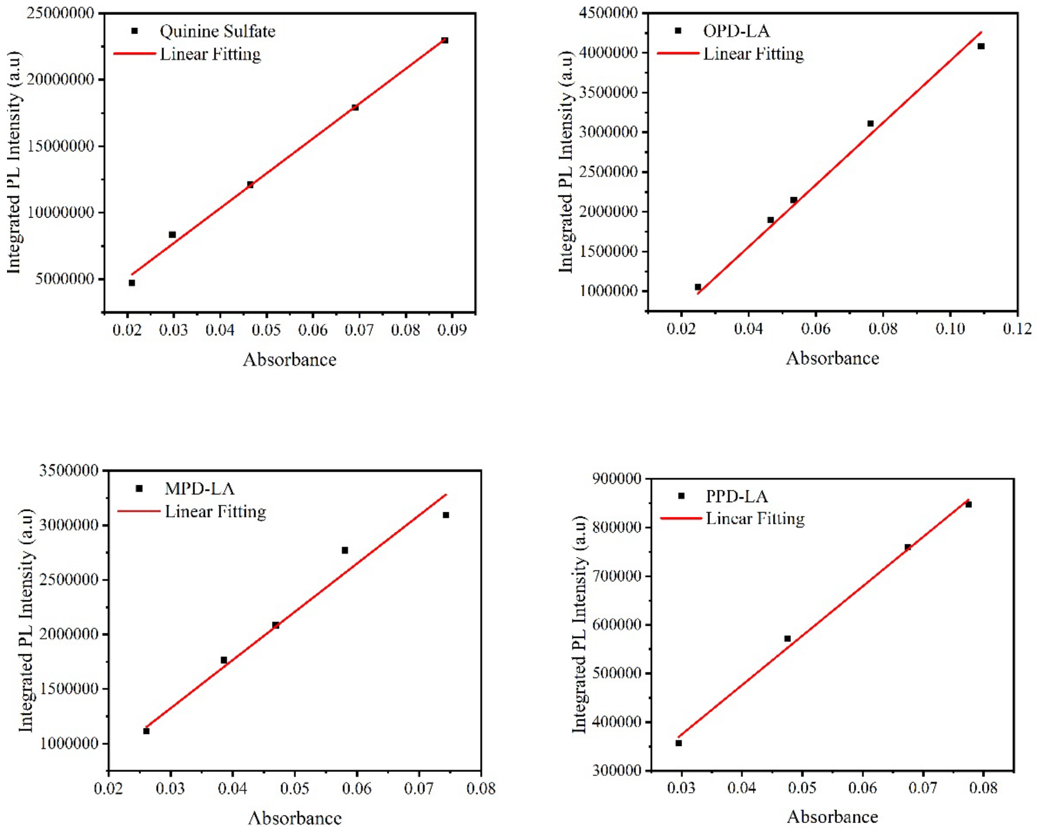

Figure S5. Plots of integrated PL intensity of OPD-LA, MPD-LA, and PPD-LA and quinine sulfate (referenced dye) as a function of optical absorbance at 365 nm and relevant data.

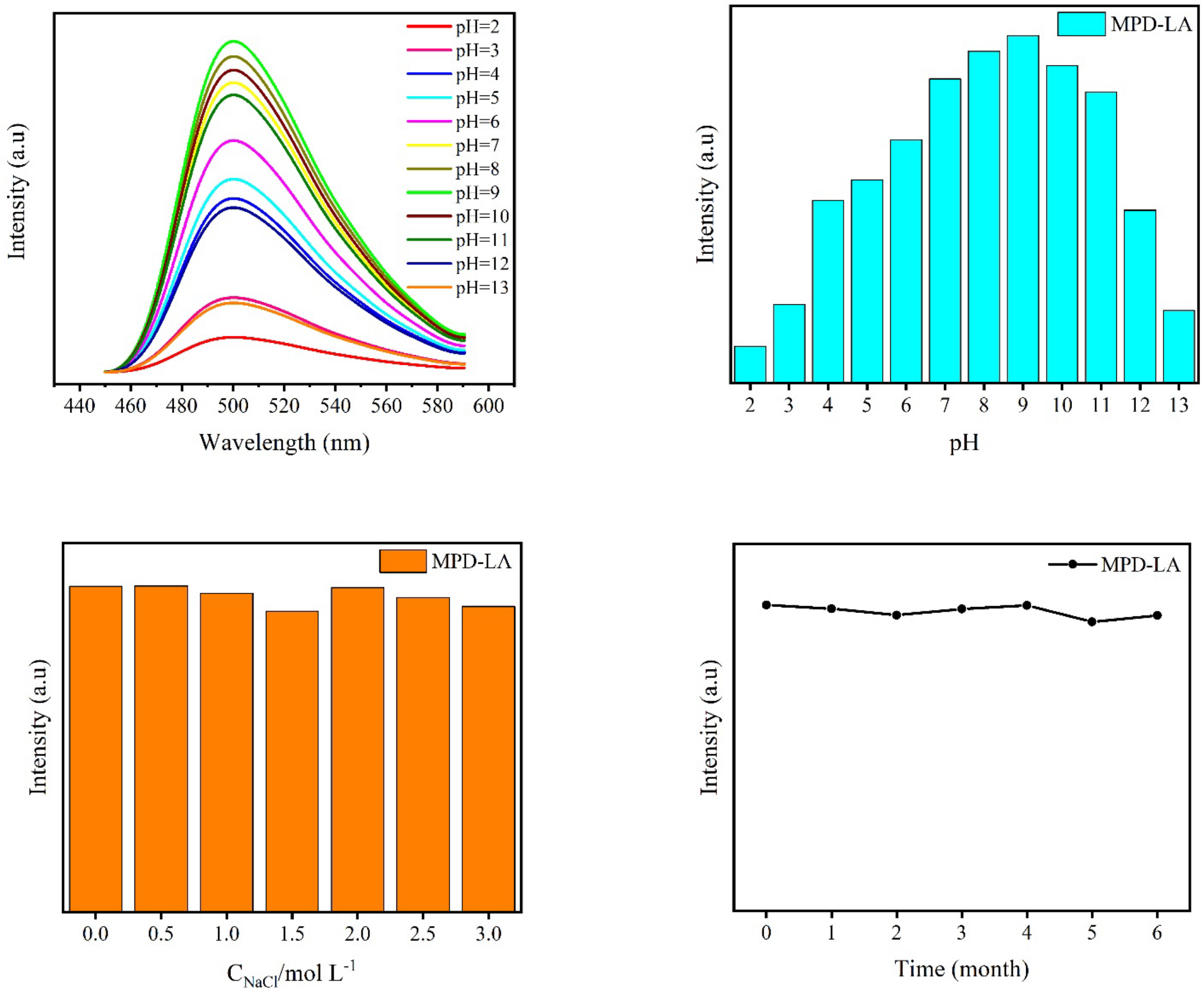

Figure S6. The fluorescence spectra and fluorescence intensity of MPD-LA at various pH values, respectively; the fluorescence intensity of MPD-LA at different NaCl concentrations; the variation in fluorescence intensity of the same MPD-LA solution at room temperature over 6 months.

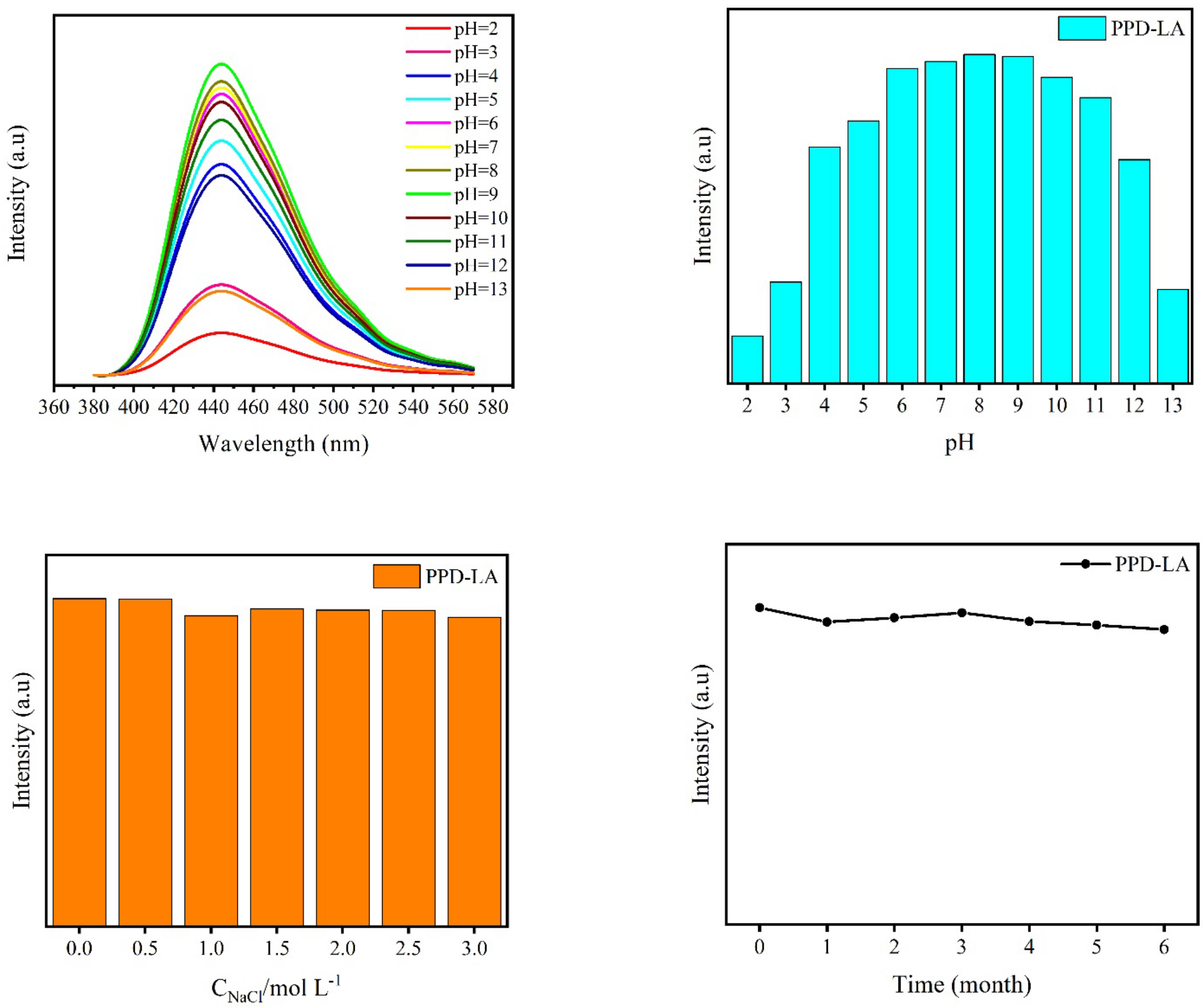

Figure S7. The fluorescence spectra and fluorescence intensity of PPD-LA at various pH values, respectively; the fluorescence intensity of PPD-LA at different NaCl concentrations; the variation in fluorescence intensity of the same PPD-LA solution at room temperature over 6 months.
